# Critical Appraisal of the Concept Frailty: Rating of Frailty in Elderly People has Weak Scientific Basis and should not be Used for Managing Individual Patients

**DOI:** 10.14336/AD.2022.0506

**Published:** 2023-02-01

**Authors:** Gunnar Akner

**Affiliations:** Geriatric Medicine, Karolinska Institutet, Stockholm, Sweden

**Keywords:** critical appraisal, concept, frailty, rating, elderly people, individual patients

## Abstract

The concept frail elderly has been used to highlight the biological, rather than chronological, age. International and national bodies recommend that individuals over age 70 who visit healthcare facilities should be screened for frailty. There are important objections to the concept. Diagnostics: ‘Frailty’ is used for several completely different types of health problems. There are no useful biomarkers, but more than 60 different published rating methods for frailty, where different methods provide very different prevalence of frailty and also do not identify the same groups of elderly people. There is significant overlap between Clinical Frailty Scale- scores and activity of daily living (ADL)-scores. There is no gold standard method against which published frailty rating scales can be validated. It is unclear when, where and how often screening for frailty should occur in healthcare. Treatment: The evidence for treatment of frailty is very weak. A recent systematic overview found that the 21 included randomised, controlled studies (RCTs) were very heterogeneous as regards inclusion/exclusion criteria, how the condition of frailty was defined, what treatment was given and what health outcomes were assessed. In addition, there are often problems with the quality of the studies. The lack of a clear definition and evidence-based treatment of frailty means that it is inappropriate to introduce assessments of frailty in individual elderly patients in health care

The concept of *frail elderly* has been used since the early 1970s [1] to highlight the biological, rather than chronological, age of elderly people, with the aim of assessing the risk of negative health outcomes at group level, as regards traits such as impaired physical functioning, including activities of daily life (ADL); falls/fractures; need for hospital care; long duration of care; institutionalisation and mortality. The scientific literature on frailty has grown extensively in the past 20 years.

The European Union recommends that all individuals over age 70 who visit healthcare facilities should be screened for frailty [2]. The International Conference of Frailty and Sarcopenia Research (ICFSR) recommends screening of all adults aged 65 and older [3-4]. Several countries have implemented screening programmes for their elderly population, including Canada [5] and Japan [6]. There has also been attempts to measure and identify frailty in adults under 60 years [7].

Despite this great interest in frailty, there are several important objections to the concept, as regards both diagnostics and treatment.

## Diagnostics

*Definition*: It has been suggested that there are several different kinds of frailty, such as physical, cognitive, social and psychological frailty [8]. This means that the umbrella term ‘frailty’ is used for four completely different types of health problems, which may or may not occur simultaneously in elderly people. There are also proposals for an integrated model for frailty [9].

*Rating scales:* There are no clinically useful biomarkers or international consensus criteria for frailty. Various research teams have tried to define frailty through operational screening/rating scales, which are sometimes binary (yes/no) and sometimes rank frailty on a scale. Two studies from 2016-2017 showed that there were at the time 67 (sic!) different published methods for assessing frailty [10-11]. Applying different frailty criteria to the same group of elderly patients has been shown to result in great variance in the prevalence of frailty, 36-88% [12] or 6-44% [13]. A 2019 report from the Swedish Agency for Health Technology Assessment and Assessment of Social Services (SBU) contains references to several other similar studies [14].

Published rating scales for frailty are based on widely differing principles: assessing autonomy/ADL, measured physical function (e.g. walking speed, timed up-and-go), questionnaires to be filled in by the patient or caregiver, a combination of physical function tests and questionnaires, data retrieved from patient records systems and more [15]. There is no way to pool the data from these different methods.

Thus, different methods of rating frailty are not only based on different principles, but also do not identify the same groups of elderly people, even if they can predict similar health outcomes at group level. The results of such assessments represent an average mathematical (statistical) risk at group level, but cannot be used to assess the risk of individual elderly people.

Claims have also been made that there are no frail elderly people; rather that the growing heterogeneity with increasing age should be described in the form of one of six frailty profiles, which gives a better basis for individualised treatment/care [16].

The 9 score Clinical Frailty Scale (CFS) is used to assess the degree of frailty [17]. The CFS criteria state that score 5 is: *“Mildly frail: These people often have more evident slowing and need help in high order iADLs”.* CSF scores 6-8 include a growing need for help with pADL. Altogether, there is significant overlap between CFS scores and ADL scores, and the question is whether an assessment of ADL capacity could replace the CFS.

*Validation:* There is no gold standard method against which the published frailty rating scales can be validated. When a frailty scale is described as ‘validated’ in the literature, this refers to predictive validation, i.e. the ability of a rating scale to predict future health outcomes and consumption of care [18], but this does not mean that they validate the concept of frailty. In addition, a large number of other health conditions can predict the same types of health outcomes. Sometimes circular evidence has been used, where particularly often cited frailty scoring methods are used as a comparison reference for other methods.

*When screening/rating should occur:* Frequently, patients are assessed for frailty at emergency wards or during hospital care. As the number of beds at hospitals has decreased, indications for admission have become so strict that most patients admitted to emergency hospitals are severely decompensated. They often have difficulties with physical function and ADL capacity, which give them high frailty scores. If frailty rating should be done, it would be reasonable to do so in elective conditions in the patient’s everyday environment (primary care), rather than during hospital care. When assessing frailty at hospital, the assessment might be of the patient’s health status two weeks prior to admission [19-20].

*How often should frailty be assessed?* The scientific literature has largely been focused on rating frailty in groups of individuals, where frailty is perceived as a dynamic process that develops over time. Longitudinal studies with repeated measures of frailty have shown upward trajectories with age with higher risk of mortality, however, with very large differences for individual people [21]. There is no basis for recommendation of how often such assessment should occur for individual people.

### Treatment

The above-mentioned consensus articles from ICFSR contain a table that summarises the current scientific knowledge about various interventions for frail older people [4, 22]. Here it is clear that there is ‘moderate’ evidence for physical activity/exercise if it is conducted in a group, but for other specified treatment measures the evidence is weak or very weak. The authors also found that there was very weak evidence for the effect of individually designed care plans for frail elderly people. The underlying extensive (93 pages!) systematic overview article included 21 randomised, controlled studies (RCTs), which were very heterogeneous as regards inclusion/exclusion criteria, how the condition of ‘frailty’ was defined, what treatment was given and what health outcomes were assessed. In addition, there are often problems with the quality of the studies [22].

It is often argued that it is potentially valuable to identify frailty in elderly individuals in order to be able to offer adequate care, which includes, among other things, a Comprehensive Geriatric Assessment (CGA). The dilemma is that CGA is not operationalised as a method, but is a recommendation for multi-domain-based diagnostics and treatment. A current review of CGA showed that there is very great variation regarding which domains are used in CGA and which specific methods are used in each domain [23]. CGA is often carried out using rating scales in various domains [24]. Another proposal is a standardised multidimensional geriatric assessment, based on a menu of rating scales, summarised in a collective risk score for negative health outcomes [25]. A Cochrane Library review from 2017 showed that a CGA carried out by a hospital for patients aged 65 and older does not affect mortality or i/p ADL capacity in the following year [26]. A recent RCT showed that systematic geriatric assessment for older (≥ 75 years) adults with frailty defined by CFS in the emergency department did not reduce hospital stay during one-year follow-up [27].

### Conclusion

The lack of a clear definition and evidence-based treatment of frailty means that it is inappropriate to introduce assessments of frailty in individual elderly patients in health care. Such an assessment can be used to create a rough basis for allocating resources at group level, but cannot and must not be used to prioritise or manage individual elderly people. A certain shift has already occurred, as frailty assessment using CFS has been used in Swedish healthcare as a basis for prioritising health care measures for individual elderly people during the early phase of the coronavirus pandemic: A policy document in the Stockholm Region recommended that elderly people living in nursing home with a CFS-score above 5 should not be admitted to hospital.

The construct ‘frailty’ is ill defined with a large amount of proposed rating scales composed by different components that define different patient populations. From a clinical standpoint, frailty research is in a premature phase and the concept should not be used in regular clinical practice for individual patients.
